# Cardiovascular Outcome in Patients Treated With SGLT2 Inhibitors for Heart Failure: A Meta-Analysis

**DOI:** 10.3389/fcvm.2021.691907

**Published:** 2021-07-14

**Authors:** Gloria M. Gager, Georg Gelbenegger, Bernd Jilma, Dirk von Lewinski, Harald Sourij, Ceren Eyileten, Krzysztof Filipiak, Marek Postula, Jolanta M. Siller-Matula

**Affiliations:** ^1^Department of Internal Medicine II, Division of Cardiology, Medical University of Vienna, Vienna, Austria; ^2^Department of Clinical Pharmacology, Medical University of Vienna, Vienna, Austria; ^3^Department of Internal Medicine, Division of Cardiology, Medical University of Graz, Graz, Austria; ^4^Department of Internal Medicine, Division of Endocrinology and Diabetology, Medical University of Graz, Graz, Austria; ^5^Department of Experimental and Clinical Pharmacology, Medical University of Warsaw, Center for Preclinical Research and Technology CEPT, Warsaw, Poland; ^6^First Chair and Department of Cardiology, Medical University of Warsaw, Warsaw, Poland

**Keywords:** SGLT2 inhibitors, clinical outcome, heart failure, pharmacotherapy, meta-analysis

## Abstract

**Background:** Sodium–glucose co-transporter 2 (SGLT2) inhibitors are an emerging class of glucose-lowering drugs that have become increasingly relevant for the treatment and prevention of heart failure (HF). Therefore, we aimed to investigate various SGLT2 inhibitors in patients with established HF at baseline and focused on the different types of HF.

**Methods:** An extensive search of PubMed and Web of Science until January 2021 was done. Two reviewers, independently and in duplicate, applied the selection criteria. This meta-analysis was conducted according to the PRISMA guidelines. Data were pooled using a random-effects model. Randomized controlled trials (RCTs) of SGLT2 inhibitors vs. a comparator in patients with HF reporting clinical outcomes were included. The primary efficacy outcome was the composite of hospitalization for HF (HHF) or cardiovascular (CV) mortality. All-cause mortality, CV mortality, and HHF were considered as secondary endpoints. Subgroup analyses involving the status of diabetes, type of HF, administered type of SGLT2 inhibitor, sex, age, body mass index (BMI), estimated glomerular filtration rate (eGFR), cause of HF, and concomitant medication were performed.

**Results:** Seventeen RCTs, comprising a total of 20,749 participants, were included (*n* = 10,848 treated with SGLT2 inhibitors and *n* = 9,901 treated with a comparator). Treatment with SGLT2 inhibitors in a HF population was associated with a 27% relative risk reduction (RRR) of HHF or CV mortality [risk ratio (RR) = 0.73, 95% CI = 0.68–0.78], 32% RRR of HHF (RR = 0.68, 95% CI = 0.62–074), 18% RRR of CV mortality (RR = 0.82, 95% CI = 0.73–0.91), and 17% RRR of all-cause mortality (RR = 0.83, 95% CI = 0.75–0.91). The effect of SGLT2 inhibitors on the primary endpoint was consistent among the different gliflozines. The effect of SGLT2 inhibitors on the primary endpoint was independent of underlying diabetes mellitus, age, sex, BMI, renal function, and HF type.

**Conclusions:** SGLT2 inhibitors are associated with improved CV outcomes in patients with HF.

## Background

Sodium–glucose co-transporter 2 (SGLT2) inhibitors are an arising drug class across antidiabetic therapeutics. During the last five years, large randomized trials have shown the cardioprotective effects of three SGLT2 inhibitors—empagliflozin, dapagliflozin, and canagliflozin—independently of the presence or absence of diabetes mellitus (DM) and within the first months after initiating the treatment (1–5). Recently, these benefits have also been demonstrated for sotagliflozin and ertugliflozin (6–8). The glucose-lowering effects of these agents are mediated through the inhibition of renal glucose reuptake in the proximal tubule of the nephron, which consequently leads to a decrease in blood glucose levels (9–11). However, it is assumed that the cardioprotective properties are of a different origin from the promoted urine glucose excretion. Although, a variety of hypotheses for SGLT2 inhibitor-induced benefits exist, the exact underlying mechanism is unclear (12). Aside from the impact on blood pressure and body weight, modulation of ion homeostasis and cellular processes are suggested (13). In particular, SGLT2 inhibitors have become progressively interesting for the treatment and prevention of heart failure (HF) (14), which has emerged as a global health issue. According to the most recent European Society of Cardiology (ESC) guidelines, HF is prevalent in 2% of the adult population and is more frequent in patients with atrial fibrillation (15, 16). Although, survival after onset of HF improved over the last 50 years (17), the 12-month all-cause mortality still occurs in about 7% of patients diagnosed with chronic HF and in 17% of patients suffering from acute HF (18–22).

Due to the promising results of the EMPA-REG OUTCOME, DECLARE-TIMI 58, CANVAS and CREDENCE trials regarding their cardiovascular (CV) outcomes in patient populations with and without HF, the next logical aim was to put focus on subgroups with an established HF at baseline, as in the DAPA-HF, EMPEROR-Reduced, and SOLOIST-WHF trials (1–6). In this respect, we performed a systematic review and meta-analysis of randomized controlled trials (RCTs) that investigated SGLT2 inhibitors in patients with established HF regarding their clinical endpoints, with a particular focus on the type of HF.

## Methods

The following systematic review and meta-analysis was conducted in accordance with the Preferred Reporting Items for Systematic Reviews and Meta-Analyses (PRISMA) guidelines, as described previously (23–28). We performed an extensive search of PubMed and Web of Science, applying predefined search terms [(empagliflozin OR dapagliflozin OR canagliflozin OR ertugliflozin OR sotagliflozin) AND heart failure AND randomized controlled trial], until January 2021. The title and abstract of suspected relevant citations were screened for eligibility, and full-text was acquired for further evaluation if the citation was deemed pertinent. The references of the retrieved meta-analyses and reviews were also examined for additional trials.

All included studies had to be RCTs, regardless of sample size, and follow-up time, comparing SGLT2 inhibitors either to placebo or a comparator and evaluated clinical endpoints. The eligible patient population for our meta-analysis comprised patients with any diagnosed HF at baseline. Two reviewers (GMG and JMSM), independently and in duplicate, applied the selection criteria. The exclusion criteria were: non-RCTs, duplicate reports, ongoing studies, studies that included patients without HF, and studies that did not assess clinical endpoints.

The primary efficacy endpoint of our meta-analysis was a composite of CV mortality or hospitalization for HF (HHF). This primary composite endpoint of HHF or CV mortality was chosen based on a uniform definition of the primary endpoint used in the majority of HF trials, which was also used in the large RCTs included into our meta-analysis. CV mortality, all-cause mortality, and HHF were considered as our secondary endpoints. For further analysis of the data, we performed subgroup analyses involving the status of diabetes, type of HF, and type of SGLT2 inhibitors used.

### Statistical Analysis

Variables are reported as number or percentages, as applicable. Risk ratios (RRs) were calculated from individual studies and pooled according to the inverse variance model with 95% confidence intervals (95% CI) and reported as relative risk reduction (RRR), as reported previously (28–32). The statistical inconsistency test (*I*^2^) was used for the assessment of any heterogeneity between the studies. We used a random-effects model for all analyses. The following sensitivity analyses were performed: (i) comparison of the results of fixed- vs. random-effects model; (ii) assessment of each study influence by successively deleting one by one to evaluate whether the pooled results of the meta-analysis change significantly; and (iii) introduction of the following subgroups: SGLT2 inhibitor type, DM vs. no DM, HF with reduced ejection fraction (HFrEF), HF with mid-range ejection fraction (HFmrEF), HF with preserved ejection fraction (HFpEF), sex, age, body mass index (BMI), estimated glomerular filtration rate (eGFR), cause of HF, and concomitant medication. We also calculated a number needed to treat (NNT) for the composite endpoint based on the mathematical formula: NNT = 1/absolute risk reduction (ARR). A two-tailed *p*-value of <0.05 was considered as significant. Review Manager (version 5.4; Copenhagen: The Nordic Cochrane Centre, The Cochrane Collaboration 2020) was used for statistical computations.

## Results

### Study Selection

Our literature search retrieved 514 references, of which 256 articles were studied more precisely based on their title or abstract (Supplementary Figure 1). The remaining references were excluded for the following reasons: non-RCTs, no clinical endpoints or not differentiating between patients with or without HF at baseline. Additionally, the retrieved reviews and meta-analyses were examined thoroughly to identify further trials, investigating the chosen topic. Seventeen trials (1–8, 33–41) were eligible for our meta-analysis, including an overall patient population of 20,749 participants, of which 10,848 patients were assigned to the SGLT2 inhibitor group and 9,901 participants were allocated to the control group (Supplementary Figure 2). The mean age of the included patient population was 67 years, whereas, the mean follow-up period comprised 18 months (ranging from 2 to 50.4 months). Our meta-analysis included three large clinical trials, which were performed only in HF participants: DAPA-HF, EMPEROR-Reduced, and SOLOIST-WHF (2, 6, 34). Furthermore, we covered data from *post-hoc* and subgroup analyses of the EMPA-REG OUTCOME (42), DECLARE-TIMI 58 (43), CANVAS (44), CREDENCE (45), VERTIS-CV (46), and SCORED (8) trials. Additionally to the regular DAPA-HF trial (2), two *post-hoc* analyses of DAPA-HF (47, 48) for our diabetes and concomitant medication subgroups were used. Furthermore, we included eight smaller studies, which were solely performed on patients with HF at baseline with or without type 2 diabetes mellitus (T2DM) (33, 35–41). Only the CANDLE Study was active comparator-controlled instead of placebo-controlled (41). The included studies are characterized in Table 1.

**Table 1:** Characteristics of the included studies.

**Study**	**Study drug**	**Study drug treatment regimen**	**Control agent**	**Study design**	**Trial participants, *n***	**Participants with HF at baseline, *n* (%)**	**Type of HF**	**Participants with T2DM at baseline, *n* (%)**	**Median follow-up**	**Median age (years)**
Zinman et al. (1)EMPA-REG OUTCOMEFitchett et al. (42)*Post*-*hoc* analysis	Empagliflozin	10 or 25 mgonce daily	Placebo	Double-blind RCT	7,020	706 (10.1)	Not specified	7,020 (100)	3.1 years	63
Dammann et al. (33)EMPA-RESPONSE-AHF	Empagliflozin	10 mgonce daily	Placebo	Double-blind RCT	79	79 (100)	Acute HF	26 (33)	60 days	76
Packer et al. (34)EMPEROR-Reduced	Empagliflozin	10 mgonce daily	Placebo	Double-blind RCT	3,730	3,730 (100)	HFrEF	1,856 (50)	16 months	67
Abraham et al. (35)EMPERIAL-Reduced	Empagliflozin	10 mgonce daily	Placebo	Double-blind RCT	312	312 (100)	HFrEF	187 (60)	12 weeks	69
Jensen et al. (36)EMPIRE-HF	Empagliflozin	10 mgonce daily	Placebo	Double-blind RCT	190	190 (100)	HFrEF	33 (17)	12 weeks	64
Mordi et al. (37)RECEDE-CHF	Empagliflozin	25 mgonce daily	Placebo	Double-blind RCT	23	23 (100)	HFrEF	23 (100)	12 weeks	70
Lee et al. (38)SUGAR-DM-HF	Empagliflozin	10 mgonce daily	Placebo	Double-blind RCT	105	105 (100)	HFrEF	82 (78)	36 weeks	69
McMurray et al. (2)DAPA-HFPetrie et al. (47)*Post*-*hoc* analysisDocherty et al. (48)*Post*-*hoc* analysis	Dapagliflozin	10 mgonce daily	Placebo	Double-blind RCT	4,744	4,744 (100)	HFrEF	2,139 (45)	1.5 years	66
Wiviott et al. (3)DECLARE-TIMI 58Kato et al. (43)*Post*-*hoc* analysis	Dapagliflozin	10 mgonce daily	Placebo	Double-blind RCT	17,160	1,724 (10)	HFrEF HF with unknown EF	17,160 (100)	4.2 years	64
Nassif et al. (39)DEFINE-HF	Dapagliflozin	10 mgonce daily	Placebo	Double-blind RCT	263	263 (100)	HFrEF	166 (63)	12 weeks	61
Singh et al. (40)REFORM	Dapagliflozin	10 mgonce daily	Placebo	Double-blind RCT	56	56 (100)	HFrEF	56 (100)	1 year	67
Neal et al. (5)CANVASRadholm et al. (44)*Post-hoc* analysis	Canagliflozin	100 or 300 mgonce daily	Placebo	Double-blind RCT	10,142	1,461 (14.4)	Not specified	10,142 (100)	3.6 years	64
Perkovic et al. (4)CREDENCESarraju et al. (45)*Post-hoc* analysis	Canagliflozin	100 mgonce daily	Placebo	Double-blind RCT	4,401	652 (15)	Not specified	4,401 (100)	2.6 years	63
Tanaka et al. (41)CANDLE	Canagliflozin	100 mgonce daily	Glimepiride	Open-label RCT	241	241 (100)	HFrEF, HFpEF	241 (100)	24 weeks	69
Cannon et al. (7)VERTIS CVCosentino et al. (46)*Post-hoc* analysis	Ertugliflozin	5 or 15 mgonce daily	Placebo	Double-blind RCT	8,246	1,958 (23.7)	Not specified	8,246 (100)	3.5 years	64
Bhatt et al. (6)SOLOIST-WHF	Sotagliflozin	200–400 mgonce daily	Placebo	Double-blind RCT	1,222	1,222 (100)	Acute HF	1,222 (100)	9 months	70
Bhatt et al. (8)SCORED	Sotagliflozin	200–400 mgonce daily	Placebo	Double-blind RCT	10,584	3,283 (31)	HFrEF, HFmrEF, HFpEF	10,584 (100)	16 months	69

*HF, heart failure; RCT, randomized controlled trial: HFrEF, HF with reduced ejection fraction; HFmrEF, HF with mid-range ejection fraction; HFpEF, HF with preserved ejection fraction*.

### Outcomes

#### Primary Composite Outcome: Hospitalization for Heart Failure or Cardiovascular Death

**Figure 1:**
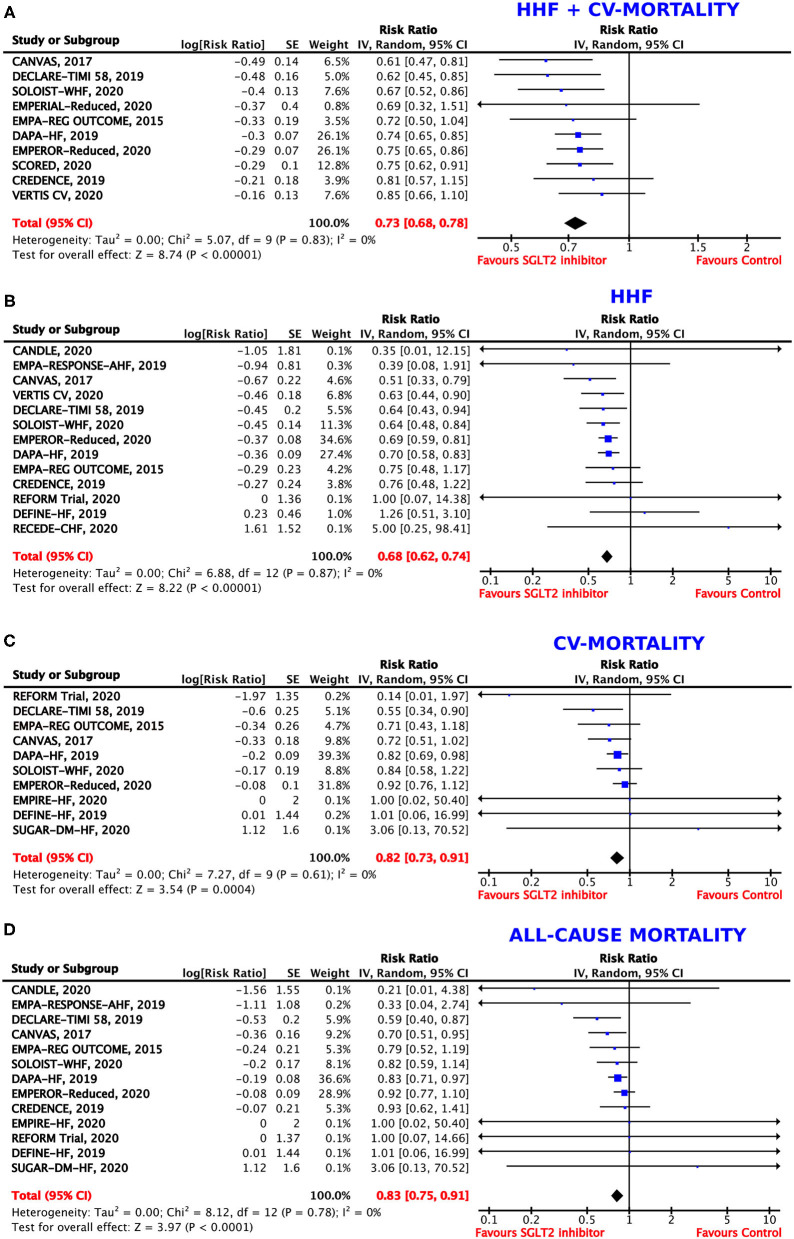
Forest plot depicting the risk ratio for **(A)** the composite outcome of hospitalization for heart failure (HHF) or cardiovascular (CV) mortality, **(B)** HHF alone, **(C)** CV mortality alone, and **(D)** all-cause mortality.

The composite outcome of HHF or CV death was regarded as our primary endpoint. Ten trials (2, 4, 6, 8, 34, 35, 42–44, 46) reported on the primary efficacy outcome. Overall, 17% of patients experienced HHF or CV death under treatment with SGLT2 inhibitors as compared to 23% in the control-group, resulting in a RRR of 27% (RR = 0.73, 95% CI = 0.68–0.78, *p* < 0.00001, *I*^2^ = 0%) (Figure 1A) and an ARR of 6%. This corresponds to a NNT of 17. For a group of 1,000 patients treated with SGLT2 inhibitors for HF for a mean time of 18 months, the composite endpoint of HHF or CV death could be prevented in 60 (Table 2).

**Table 2:** Incidence, relative risk reduction (RRR), absolute risk reduction (ARR), and number needed to treat (NNT) for the primary composite endpoint and secondary endpoints.

	**Event**	**Incidence of SGLT2 inhibitor (%)**	**Incidence of comparator (%)**	**RRR (%)**	**ARR (%)**	**NNT**	**No. of events reduced with SGLT2 inhibitors per 1,000 treated patients**	***p*-value**
	Primary composite outcome: HHF or CV mortality	17	23	27	6	17	60	** <0.001**
	HHF	11	16	32	5	20	50	** <0.001**
	CV mortality	9	11	18	2	50	20	** <0.001**
	All-cause mortality	11	13	17	2	50	20	** <0.001**
**Main analyses**								
Diabetes	Primary composite outcome: HHF or CV mortality	18	25	28	7	15	70	** <0.001**
Non-Diabetes		14	18	24	4	25	40	** <0.001**
HFrEF (EF ≤ 40%)	Primary composite outcome: HHF or CV mortality	18	24	25	6	17	60	** <0.001**
HFrEF (EF ≤ 45%)		18	29	38	11	10	110	**0.003**
HFmrEF (EF = 40–50%)		18	32	42	14	8	140	**0.003**
HFpEF (EF > 45%)		14	18	21	4	25	40	0.18
HFpEF (EF > 50%)		13	18	30	5	20	50	**0.01**
Acute HF		40	59	33	18	6	180	**0.002**
Unknown EF		14	19	26	5	20	50	** <0.001**
Canagliflozin	Primary composite outcome: HHF or CV mortality	14	21	31	7	15	70	**0.007**
Dapagliflozin		15	21	28	6	17	60	** <0.001**
Empagliflozin		17	23	26	5	20	50	** <0.001**
Ertugliflozin		12	14	15	2	50	20	0.22
Sotagliflozin		40	56	28	16	7	160	** <0.001**
Canagliflozin	HHF	6	10	39	4	25	40	**0.002**
Dapagliflozin		10	14	30	4	25	40	** <0.001**
Empagliflozin		13	17	30	4	25	40	** <0.001**
Ertugliflozin		5	8	37	3	34	30	**0.01**
Sotagliflozin		31	49	36	18	6	180	**0.001**
Canagliflozin	CV mortality	9	12	28	3	34	30	0.07
Dapagliflozin		8	12	29	4	25	40	**0.04**
Empagliflozin		9	10	10	1	100	10	0.24
Sotagliflozin		8	10	16	2	50	20	0.37
Canagliflozin	All-cause mortality	10	13	23	3	34	30	**0.04**
Dapagliflozin		11	14	21	3	34	30	**0.001**
Empagliflozin		12	13	10	1	100	10	0.19
Sotagliflozin		11	13	18	2	50	20	0.24
**Subgroup analyses**								
Male	Primary composite outcome: HHF or CV mortality	15	20	26	5	20	50	** <0.001**
Female		11	16	30	5	20	50	** <0.001**
Age <65 years		15	20	25	5	20	50	** <0.001**
Age ≥ 65 years		14	19	28	5	20	50	** <0.001**
BMI <30 kg/m^2^		16	22	26	6	17	60	** <0.001**
BMI ≥ 30 kg/m^2^		17	23	27	6	17	60	** <0.001**
eGFR 30–60 (ml/min/1.73 m^2^)		15	20	27	5	20	50	** <0.001**
eGFR ≥60 (ml/min/1.73 m^2^)		13	18	27	5	20	50	** <0.001**
Ischemic HF		16	22	27	6	17	60	**0.001**
Non-Ischemic HF		15	21	29	6	17	60	** <0.001**
Use of MRA		15	21	27	6	17	60	** <0.001**
No use of MRA		14	18	25	4	25	40	** <0.001**
Use of ARNI		16	19	16	3	34	30	0.31
No use of ARNI		16	22	27	6	17	60	** <0.001**

*SGLT2, sodium-glucose co-transporter 2; RRR, relative risk reduction; ARR, absolute risk reduction; NNT, number needed to treat; HFrEF, heart failure with reduced ejection fraction; CV, cardiovascular; HF, heart failure; EF, ejection fraction; HHF, hospitalization for HF; BMI, body mass index; eGFR, estimated glomerular filtration rate; MRA, mineralocorticoid receptor antagonist; ARNI, angiotensin receptor neprilysin inhibitor. Bold values are deemed as statistically significant*.

#### Hospitalization for Heart Failure

Thirteen trials (2, 6, 33, 34, 37, 39–41, 43–46, 49) provided data on the incidence of HHF. In the patient population assigned to SGLT2 inhibitors, 11% experienced HHF. In contrast, 16% patients who were allocated to the control group were hospitalized due to HF. SGLT2 inhibitor use therefore resulted in a RRR of HHF by 32% (RR = 0.68, 95% CI = 0.62–0.74, *p* < 0.00001, *I*^2^ = 0%) (Figure 1B) and an ARR of 4%.

#### Cardiovascular Mortality

Overall, 10 trials reported on CV mortality (2, 6, 34, 36, 38–40, 43, 44, 49). Treatment with SGLT2 inhibitors was associated with a RRR of 18% to die from CV causes (RR = 0.82, 95% CI = 0.73–0.91, *p* < 0.001, *I*^2^ = 0%) (Figure 1C) and an ARR of 2%. When treated with SGLT2 inhibitors, CV mortality occurred in 9% of patients as compared to 11% of patients allocated to the comparison group.

#### All-Cause Mortality

In patients treated with SGLT2 inhibitors, all-cause mortality was reported in 11% as compared to 13% in patients treated with placebo or a comparator. Consequently, the RRR to die from any cause was 17% (RR = 0.83, 95% CI = 0.75–0.91, *p* < 0.001, *I*^2^ = 0%) (Figure 1D) with an ARR of 2%, when assigned to SGLT2 inhibitors.

### Main Analyses for the Primary Composite Endpoint

#### Status of Diabetes Mellitus

**Figure 2:**
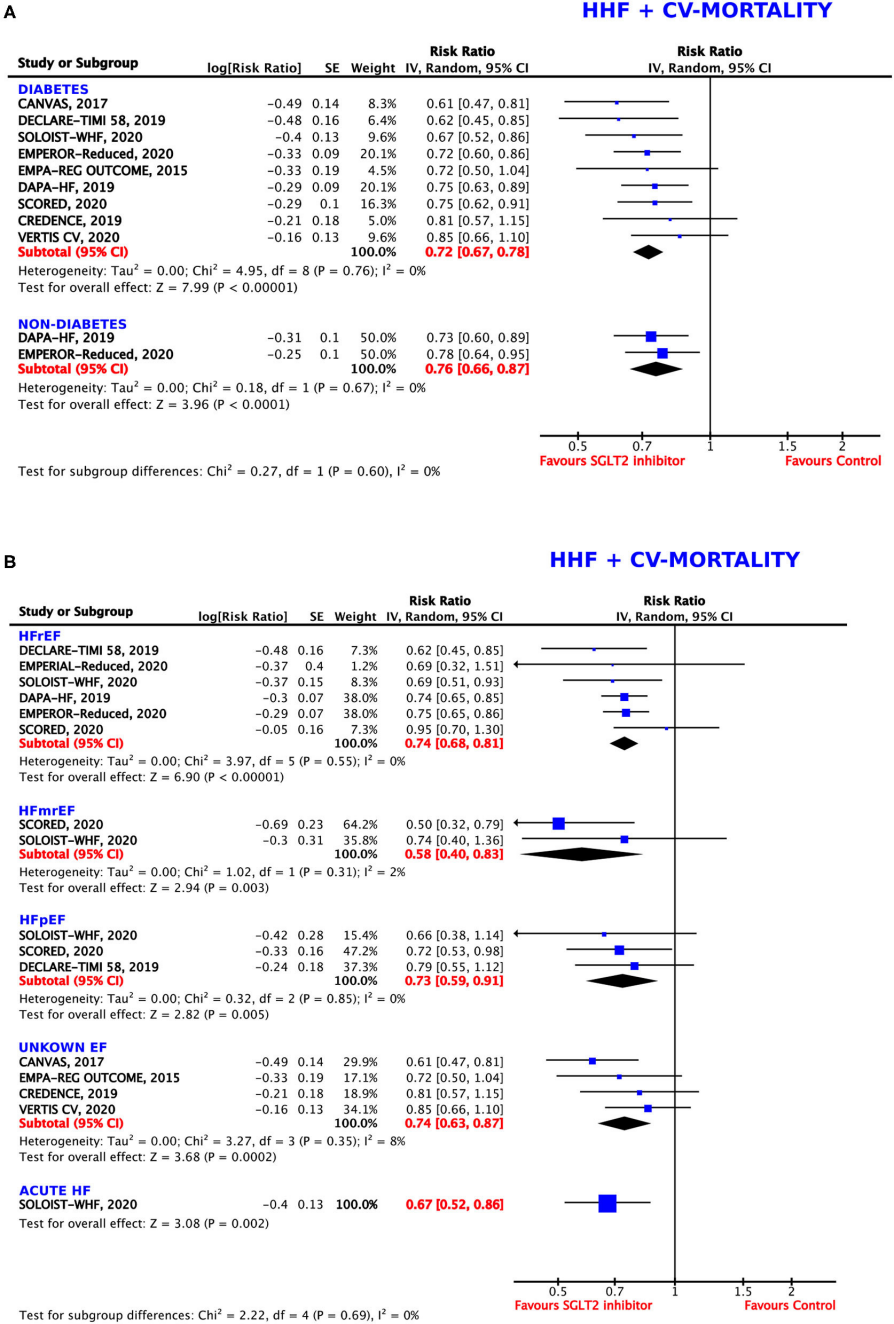
Forest plot depicting the relative risk (RR) for the composite outcome of hospitalization for heart failure (HHF) or cardiovascular (CV) mortality in **(A)** patients with or without diabetes and **(B)** depending on the type of heart failure (HF): HFrEF (HF with reduced ejection fraction), HFmrEF (HF with mid-range ejection fraction), HFpEF (HF with preserved ejection fraction), unknown EF, and acute HF.

We investigated the composite endpoint of HHF or CV death in populations with and without DM. Both groups showed statistically significant results for treatment with SGLT2 inhibitors, but the magnitude of the effect was numerically, albeit not statistically, larger in patients with DM (RRR = 28%, RR = 0.72, 95% CI = 0.67–0.78, *p* < 0.00001, *I*^2^ = 0%) as compared to patients without DM (RRR = 24%, RR = 0.76, 95% CI = 0.66–0.87, *p* < 0.0001, *I*^2^ = 0%, *p*-value for subgroup differences = 0.60) (Figure 2A), with an ARR of 7%.

#### Type of Heart Failure

Subgroup analysis for the composite endpoint was also performed considering the type of HF (Figure 2B). SGLT2 inhibitors worked comparably well in patients diagnosed with HFrEF (EF ≤40% and ≤ 45%, respectively), with a resulting RRR of 26% (RR = 0.74, 95% CI = 0.68–0.81, *p* < 0.00001, *I*^2^ = 0%), in patients with HFpEF (EF >45% and >50%, respectively), with a RRR of 27% (RR = 0.73, 95% CI = 0.59–0.91, *p* = 0.005, *I*^2^ = 0%), and in patients with an unknown (not specified) EF, with a RRR of 26% (RR = 0.74, 95% CI = 0.63–0.87, *p* = 0.0002, *I*^2^ = 8%). In patients with acute HF, SGLT2 inhibitors were even more beneficial (RRR = 33%, RR = 0.67, 95% CI = 0.52–0.86, *p* = 0.002). The greatest effects were seen in participants diagnosed with HFmrEF (EF = 40–50%), leading to a RRR of 42% (RR = 0.58, 95% CI = 0.40–0.83, *p* = 0.003, *I*^2^ = 2%).

The differences between the individual subgroups were not deemed statistically significant (*p* = 0.69).

### Analyses for Type of SGLT2 Inhibitor

#### Primary Composite Outcome: Hospitalization for Heart Failure or Cardiovascular Death

**Figure 3:**
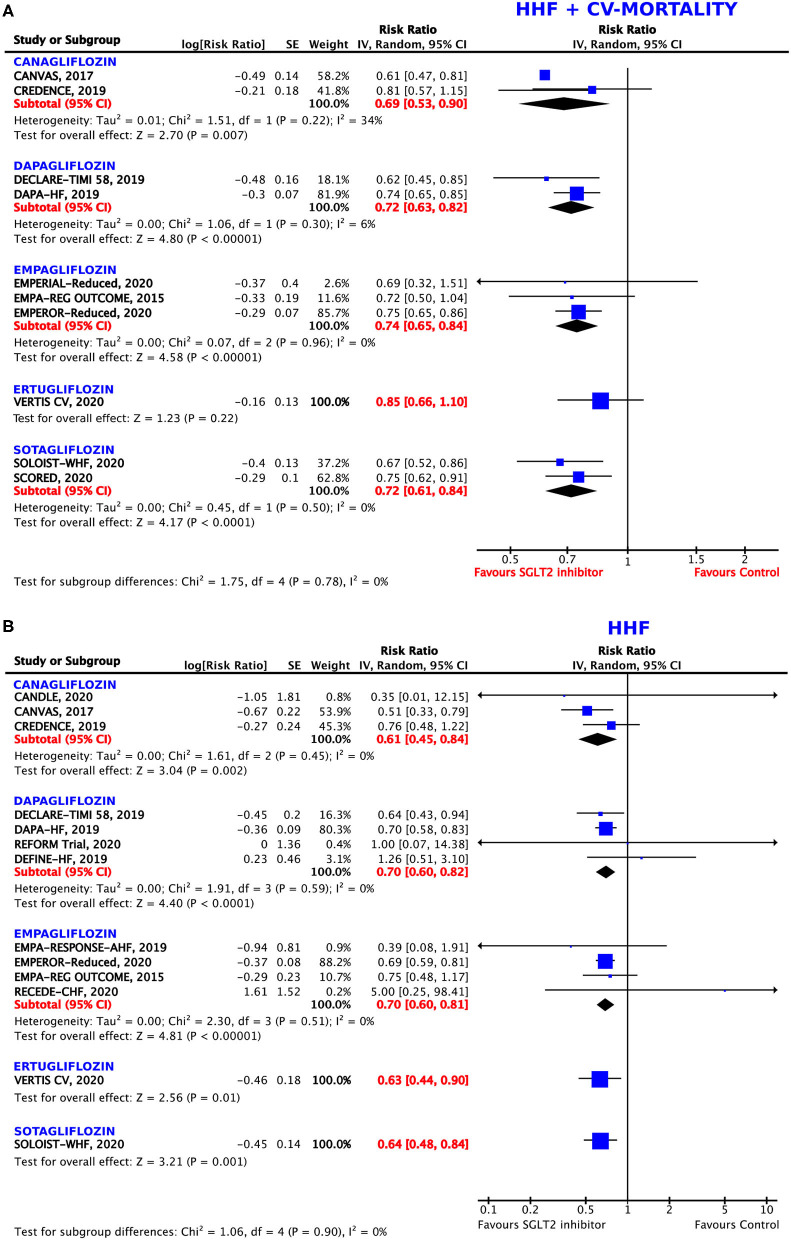
Forest plot depicting the relative risk (RR) according to the administered type of sodium–glucose co-transporter 2 (SGLT2) inhibitor for **(A)** the composite outcome of hospitalization for heart failure (HHF) or cardiovascular (CV) mortality and **(B)** HHF alone.

The direction of the effect of SGLT2 inhibition on the composite endpoint was comparable for all five agents (Figure 3A). The magnitude of the effect was similar between dapagliflozin (RRR = 28%, RR = 0.72, 95% CI = 0.63–0.82, *p* < 0.00001, *I*^2^ = 6%), sotagliflozin (RRR = 28%, RR = 0.72, 95% CI = 0.61–0.84, *p* < 0.0001, *I*^2^ = 0%), and empagliflozin (RRR = 26%, RR = 0.74, 95% CI = 0.65–0.84, *p* < 0.00001, *I*^2^ = 0%). Canagliflozin was associated with the highest RRR of 31% as compared to the control arm (RR = 0.69, 95% CI = 0.53–0.90, *p* = 0.007, *I*^2^ = 34%) in *post-hoc* analyses of the RCTs. Ertugliflozin missed statistical significance (*p* = 0.22).

#### Hospitalization for Heart Failure

All five agents reached significant values for the prevention of HHF as compared to the control arm (Figure 3B). Again, canagliflozin was demonstrated to have the greatest effects on this endpoint, with a RRR of 39% (RR = 0.61, 95% CI = 0.45–0.84, *p* = 0.002, *I*^2^ = 0%), followed by ertugliflozin (RRR = 37%, RR = 0.63, 95% CI = 0.44–0.90, *p* = 0.01) and sotagliflozin (RRR = 36%, RR = 0.64, 95% CI = 0.48–0.84, *p* = 0.001). The magnitude of the effect was similar between dapagliflozin (RRR = 31%, RR = 0.69, 95% CI = 0.57–0.85, *p* = 0.0004, *I*^2^ = 8%), and empagliflozin (RRR = 30%, RR = 0.70, 95% CI = 0.60–0.81, *p* < 0.00001, *I*^2^ = 0%).

#### Cardiovascular Mortality

**Figure 4:**
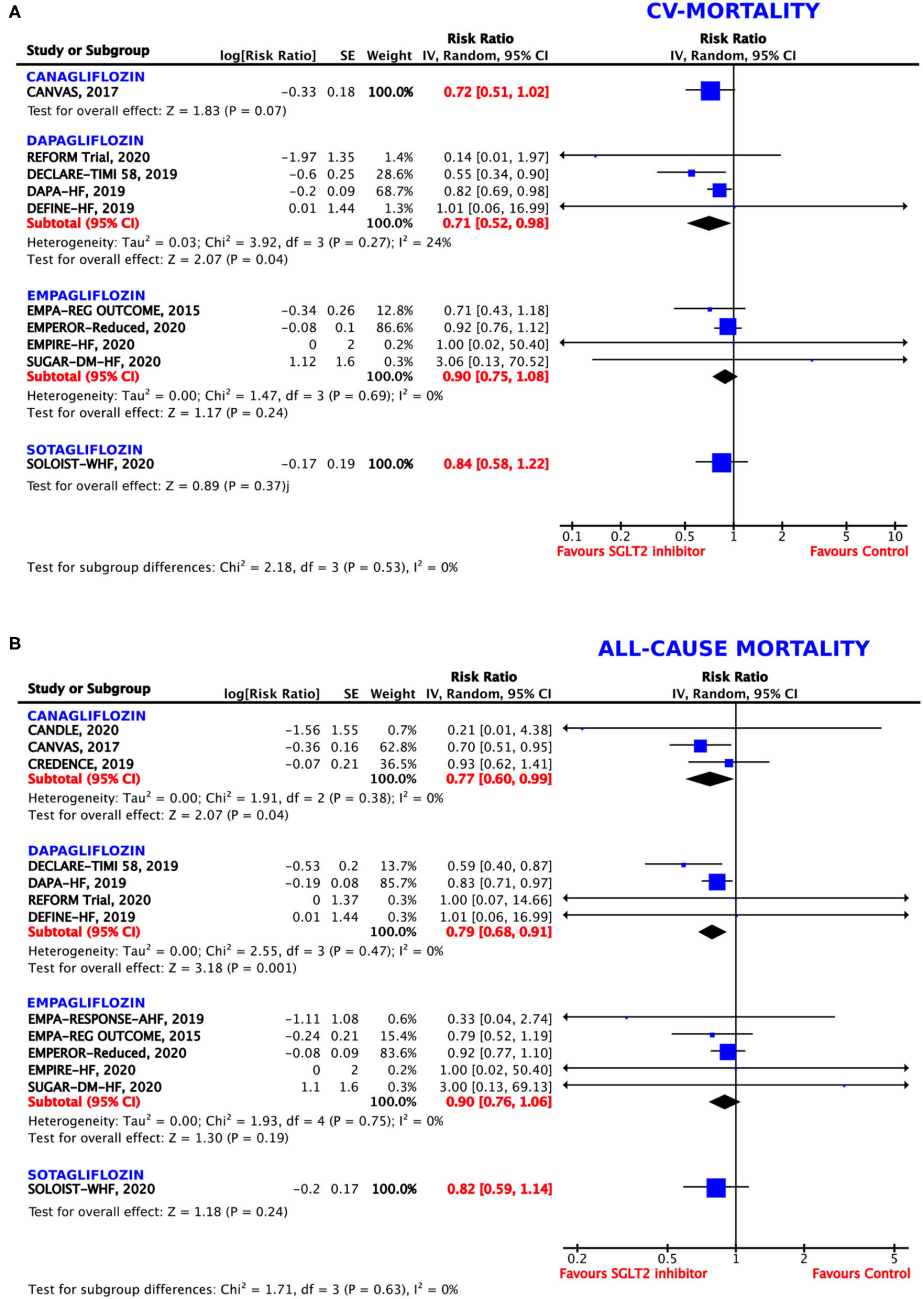
Forest plot depicting the relative risk (RR) according to the administered type of SGLT2 inhibitor for **(A)** cardiovascular (CV) mortality alone and **(B)** all-cause mortality.

Although, the direction of the effect was similar for all the tested agents, empagliflozin, canagliflozin, and sotagliflozin did not reach statistical significance for the reduction of CV mortality (*p* = 0.24, 0.07, and 0.37, respectively). Dapagliflozin showed borderline significance, obtaining a 29% RRR of death from CV causes (RR = 0.71, 95% CI = 0.52–0.98, *p* = 0.04, *I*^2^ = 24%) (Figure 4A).

#### All-Cause Mortality

Although, the direction of the effect was similar for all the tested agents, the magnitude of the benefit differed between the different SGLT2 inhibitors (Figure 4B). Canagliflozin showed a RRR of 23% (RR = 0.77, 95% CI = 0.60–0.99, *p* = 0.04, *I*^2^ = 0%) and dapagliflozin a RRR of 21% (RR = 0.79, 95% CI = 0.79–0.91, *p* = 0.001, *I*^2^ = 55%) for all-cause mortality. However, empagliflozin and sotagliflozin did not reach statistical significance for the reduction of all-cause mortality (*p* = 0.19 and 0.24, respectively).

### Subgroup Analyses for the Primary Composite Outcome According to Patients' Baseline Data

#### Sex

**Figure 5:**
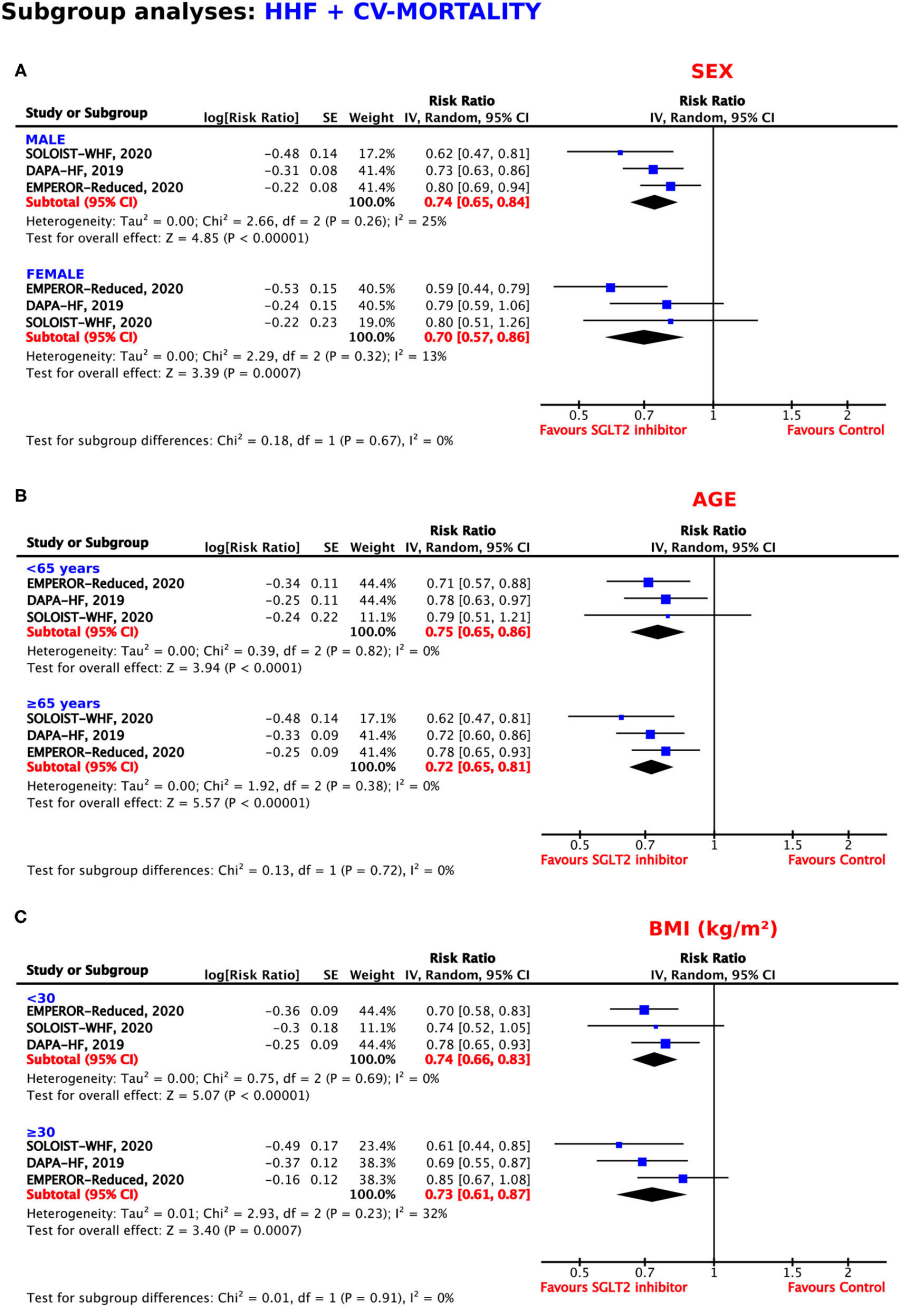
Subgroup analyses for the composite outcome according to **(A)** sex, **(B)** age, and **(C)** body mass index.

Both men and women profited from treatment with SGLT2 inhibitors. As shown in Figure 5A, these benefits were even more pronounced in the female population, with a resulting RRR of 30% (RR = 0.70, 95% CI = 0.57–0.86, *p* = 0.0007, *I*^2^ = 13%) as compared to a RRR of 26% in male patients (RR = 0.74, 95% CI = 0.65–0.84, *p* < 0.00001, *I*^2^ = 25%).

#### Age

As shown in Figure 5B, the SGLT2 inhibitors reached statistical significance in patients <65 years of age and in patients 65 years or older for the reduction of the composite endpoint, with RRRs of 25% and 28%, respectively (RR = 0.75, 95% CI = 0.65–0.86, *p* < 0.0001, *I*^2^ = 0%; RR = 0.72, 95% CI = 0.65–0.81, *p* < 0.00001, *I*^2^ = 0%).

#### BMI (kg/m^2^)

The magnitude of the effect for the prevention of HHF or CV mortality was similar in patients with a BMI <30 and a BMI ≥30 kg/m^2^ (RRR = 26%, RR = 0.74, 95% CI = 0.66–0.83, *p* < 0.00001, *I*^2^ = 0%; RRR = 27%, RR = 0.73, 95% CI = 0.61–0.87, *p* = 0.0007, *I*^2^ = 32%) (Figure 5C).

#### eGFR (ml/min/1.73 m^2^)

**Figure 6:**
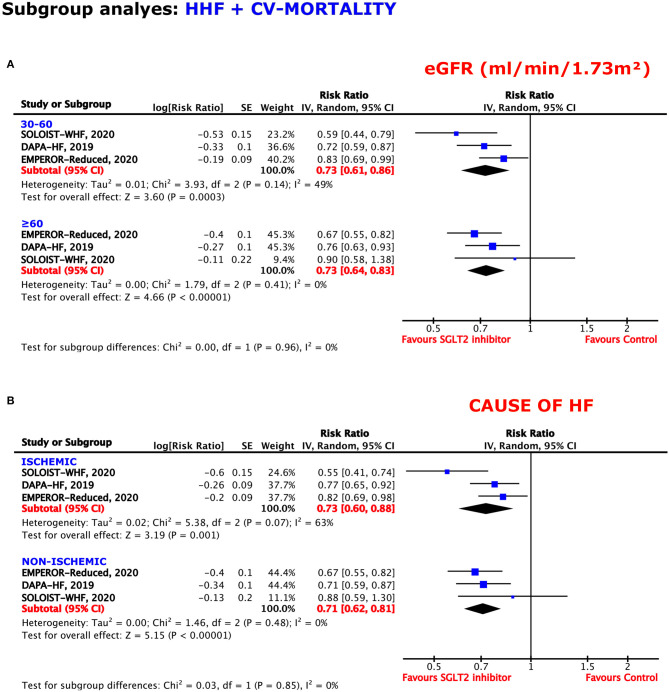
Subgroup analyses for the composite outcome according to the **(A)** estimated glomerular filtration rate (eGFR) and **(B)** cause of HF.

The benefit of SGLT2 inhibition was independent of patients' eGFRs, obtaining a RRR of 27% in both patients with eGFRs of 30–60 and ≥60 (RR = 0.73, 95% CI = 0.61–0.86, *p* = 0.0003, *I*^2^ = 49%; RR = 0.73, 95% CI = 0.64–0.83, *p* < 0.00001, *I*^2^ = 0%) (Figure 6A).

#### Cause of HF

Regardless of an ischemic or non-ischemic origin of HF, SGLT2 inhibition reached sufficient effects for the prevention of the composite endpoint. When suffering from ischemic HF, the resulting RRR obtained was 27%, whereas, the RRR was 29% when a non-ischemic HF was diagnosed (RR = 0.73, 95% CI = 0.60–0.88, *p* = 0.001, *I*^2^ = 63%; RR = 0.71, 95% CI = 0.62–0.81, *p* < 0.00001, *I*^2^ = 0%) (Figure 6B).

#### Use of Mineralocorticoid Receptor Antagonists

**Figure 7:**
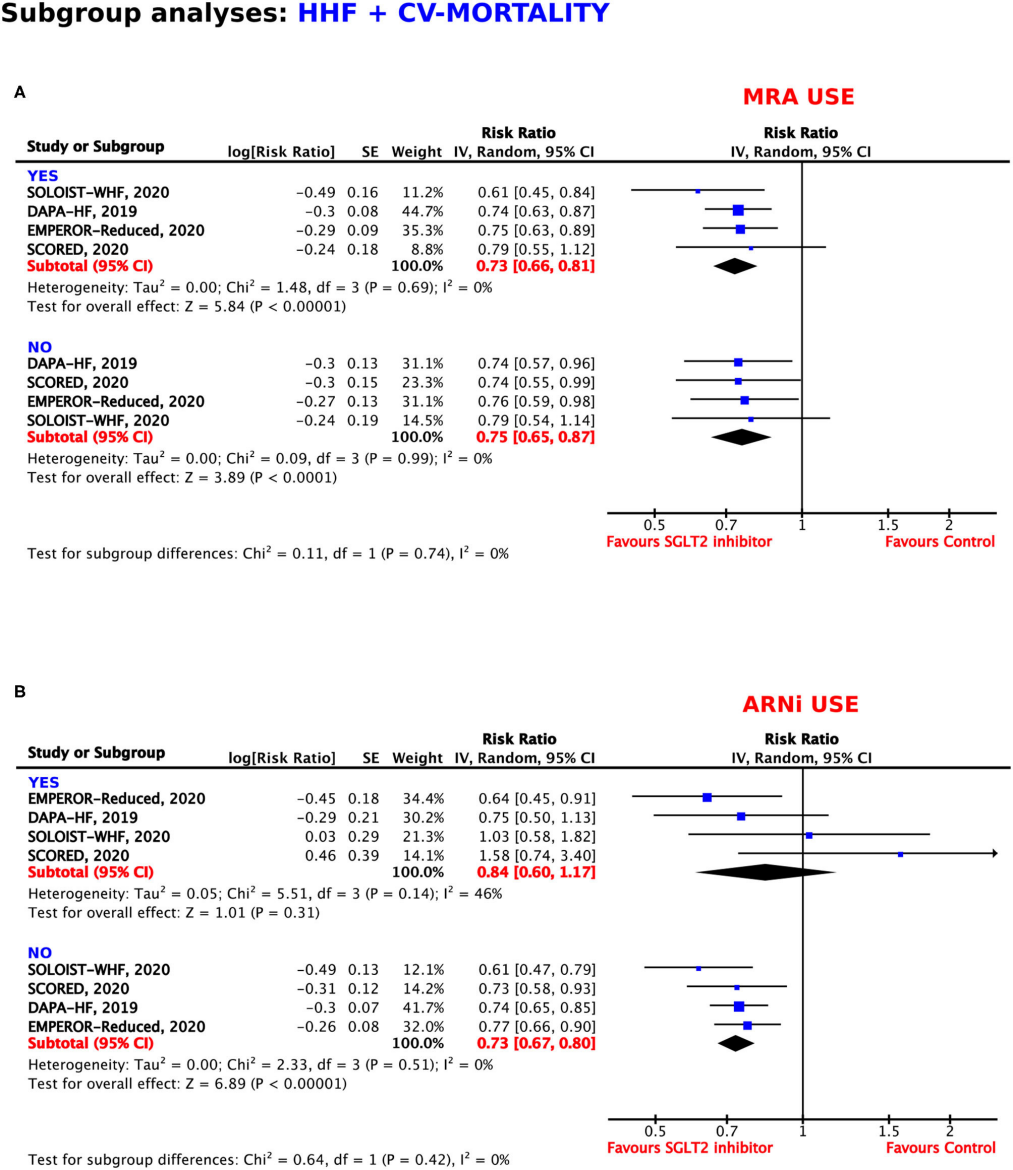
Subgroup analyses for the composite outcome according to concomitant use of **(A)** mineralocorticoid receptor antagonists (MRAs) and **(B)** angiotensin receptor neprilysin inhibitors (ARNIs).

Concomitant medication with mineralocorticoid receptor antagonists (MRAs) did not modify the beneficial effects of treatment with SGLT2 inhibitors. The use of MRAs was associated with a RRR of 27% (RR = 0.73, 95% CI = 0.66–0.81, *p* < 0.00001, *I*^2^ = 0%), whereas, renunciation of MRA use obtained a RRR of 25% (RR = 0.75, 95% CI = 0.65–0.87, *p* < 0.00001, *I*^2^ = 0%) (Figure 7A).

#### Use of Angiotensin Receptor Neprilysin Inhibitors

In patients under treatment with angiotensin receptor neprilysin inhibitors (ARNIs), the RRR of SGLT2 inhibition was 16%, which did not reach statistical significance (*p* = 0.31). In comparison, SGLT2 inhibitor treatment resulted in a RRR of 27% in patients who were also treated with ARNIs (RR = 0.73, 95% CI = 0.67–0.80, *p* < 0.00001, *I*^2^ = 0%) (Figure 7B).

#### Period of Follow-Up (≤1.5 and >1.5 Years)

Based on the mean follow-up time of 18 months, we set the cutoff for the subgroup analysis at 1.5 years (≤1.5 and >1.5 years; Supplementary Figure 3). The shorter follow-up period (≤1.5 years) did not alter the beneficial effects negatively, which were observed under treatment with SGLT2 inhibitors, with a resulting RRR of 26% (RR = 0.74, 95% CI = 0.68–0.80, *p* < 0.00001, *I*^2^ = 0%). In comparison, follow-up times longer than 1.5 years were associated with a RRR of 28% (RR = 0.72, 95% CI = 0.62–0.83, *p* < 0.00001, *I*^2^ = 8%). Set side by side to the overall analysis for the composite endpoint with a RRR of 27%, the effects are of similar magnitude (RR = 0.73, 95% CI = 0.68–0.78, *p* < 0.00001, *I*^2^ = 0%).

#### Sensitivity Analysis

The results of the random and fixed effects were revealed to be similar (data not shown).

Excluding single studies from the analyses did not change the direction and the magnitude of the effect. In patients who were treated with ARNIs, the use of sotagliflozin did not result in a reduction of the primary endpoint, but the use of dapagliflozin or empagliflozin was associated with a reduction of the primary endpoint.

The visual inspection of the funnel plot has shown minor asymmetry (data not shown).

## Discussion

The present meta-analysis investigated various RCTs of SGLT2 inhibitors regarding their clinical outcomes, with a particular focus on a patient population with established HF at baseline. Our results highlight that all SGLT2 inhibitors lower the risk of CV death, all-cause mortality, and HHF (1–3, 5) in patients with underlying HF diagnosis. Hospitalization for HF emerged as the outcome, which was best reduced by SGLT2 inhibitors (RRR = 32%).

Previous meta-analyses have already reported on the outstanding ability of different SGLT2 inhibitors in decreasing the rates of hospitalization due to heart failure in participants with or without HF at baseline (50, 51). In our analysis, we put focus on the different types of HF, including HFrEF (EF ≤40% and ≤45%), HFmrEF (EF 40–50%), HFpEF (EF >45% and >50%), and acute HF. We also included a subgroup of HF with unknown EF due to the fact that some studies did not classify between the different forms of HF, such as the EMPA-REG OUTCOME, CANVAS, CREDENCE, or VERTIS-CV *post-hoc* analysis (44–46, 49). Furthermore, some studies solely focused on patients with HFrEF [EMPEROR-Reduced (34), DAPA-HF trial (2), and DEFINE-HF trial (39)] or used different left ventricular ejection fraction (LVEF) thresholds to distinguish between HFrEF and HFpEF [DECLARE-TIMI 58 *post-hoc* analysis, EF < 45% (43); CANDLE, SOLOIST WHF, and SCORED trial, EF < 50% (6, 8, 41)]. In addition, the CANDLE trial did not assess the clinical safety endpoints with regard to the baseline LVEF (41). The reduction of HHF or CV mortality was of similar magnitude in most groups. Interestingly, our analysis show that SGLT2 inhibitors are also effective in HFmrEF and HFpEF, which is a novel finding as there is currently no effective therapy in this patient population. Nevertheless, the majority of the data for the HFmrEF and HFpEF groups were based on trials using sotagliflozin. Therefore, the question remains as to whether SGLT2 inhibitors display a class effect in this unique patient population.

The standard treatment approach for HFrEF currently includes angiotensin-converting enzyme (ACE) inhibitors, MRAs, and beta-blockers. ARNIs, I_f_-channel inhibitors, and diuretics are also often administered in HFrEF. To date, no pharmacotherapy ameliorating the prognosis in patients with HFpEF has been proven; therefore, treatment of the symptoms and comorbidities is the current approach (52–54). Although, some data indicate SGLT2 inhibitors as a sufficient therapy option for patients with HFpEF, results of large outcome trials, which investigate particularly participants with HFpEF, such as the EMPEROR-Preserved trial (Empagliflozin Outcome Trial in Patients with Chronic Heart Failure with Preserved Ejection Fraction; NCT03057951) or the DELIVER trial (Dapagliflozin Evaluation to Improve the Lives of Patients with Preserved Ejection Fraction Heart Failure; NCT03619213), are ultimately required to confirm these assumptions (55, 56).

Heart failure is highly associated with comorbidities, which directly affect patients' mortality and morbidity. A survey from 2014 demonstrated that 74% of 3,226 patients with chronic HF suffer at least from one comorbidity, the most frequent being chronic kidney disease, anemia, and diabetes (57). The link between HF and diabetes has been reported previously (58), making medication treating both conditions even more appealing. Interestingly, a *post-hoc* analysis of the DAPA-HF trial (2) showed that dapagliflozin acted comparably efficiently in reducing the composite endpoint in individuals with or without diabetes (HR = 0.75 vs. 0.73, *p*-value for interaction = 0.83). According to these results, one can assume that dapagliflozin is applicable to patients with HFrEF, irrespective of their glycemic status (34). Of note is that dapagliflozin indeed reduced the glycated hemoglobin (HbA1c) in people with diabetes; however, it had no effect on HbA1c in patients without diagnosed diabetes (47). The safety and tolerability of dapagliflozin in people without diabetes were also suggested in the DEFINE-HF trial; nevertheless, this study was not powered for clinical outcomes, including a small sample size of only 263 patients (63% of which had T2DM) and a short follow-up period of only 12 weeks (39). The very recent EMPEROR-Reduced trial also demonstrated a similar efficacy in reducing the composite endpoints of CV mortality and HHF in the diabetic and non-diabetic subpopulations (HR = 0.72 vs. 0.78), leading to the assumption that empagliflozin is also beneficial in people with HFrEF independent of the presence of diabetes (34). Our meta-analysis further confirmed the similar efficacy profiles of canagliflozin, dapagliflozin, empagliflozin, and sotagliflozin for their composite endpoint, regardless of the diabetes status.

Cardiovascular mortality and all-cause mortality were also reduced in patients who were administered SGLT2 inhibitors as compared to the control group, with a RRR of 17–18%. Interestingly, when analyzed with regard to the type of SGLT2 inhibitor used (empagliflozin, dapagliflozin, canagliflozin, and sotagliflozin), the magnitude of the effect varied, which might be due to the differences in the studied populations. Interestingly, dapagliflozin reduced CV mortality in our meta-analysis in HF patients significantly, whereas, empagliflozin, canagliflozin, and sotagliflozin did not reach statistical significance. Importantly, a recent meta-analysis that investigated SGLT2 inhibitors for CV outcomes in patients with T2DM independently of HF diagnosis demonstrated a superiority of empagliflozin as compared to dapagliflozin and canagliflozin in preventing all-cause and CV mortality (59). However, these differences might be based on the differing study populations, with our analysis particularly focusing on patients with HF at baseline rather than on the diabetes status. Further trials addressing the question of superiority within the SGLT2 inhibitor class are warranted.

We would like to put a special emphasis on the very low NNT of 17 for the composite outcome, which we have shown for the SGLT2 inhibitors in our meta-analysis. For comparison, in 2014, the PARADIGM-HF trial demonstrated a NNT of 32 for the prevention of CV mortality in patients with HF when treated with ARNIs, which are nowadays indispensable for treating HF (60). Of note is that our subgroup analysis showed no benefit for the concomitant use of SGLT2 inhibitors and ARNIs. However, that outcome was solely generated by the missing advantage of sotagliflozin use in addition to ARNI treatment in the SOLOIST-WHF and SCORED trials (6, 8), while the combination of dapagliflozin or empagliflozin with ARNIs resulted in beneficial effects, as demonstrated in a recent meta-analysis (61). Whether sotagliflozin constitutes as just an exception or whether these results were based on differences in the study population remains unclear. However, current data support the combined administration of these drug classes in patients with HF (62).

Hence, once more, our current meta-analysis emerges as highly relevant due to further confirming and demonstrating the outstanding properties of SGLT2 inhibitors in HF management in patients already on standard-of-care treatment.

## Strengths of This Meta-Analysis and Differences as Compared to Other Meta-Analyses

The most distinctive difference between our meta-analysis and other recent ones (63, 64) is that this is the first meta-analysis investigating all current RCTs on SGLT2 inhibitors on HF with clinical endpoints, irrespective of the size of the trial. Furthermore, we included all available data of *post-hoc* analyses from large trials to elaborate the safety and efficacy of SGLT2 inhibitors in patients with established HF at baseline, irrespective of their diabetes status. In addition we also focused on subgroups, especially the different types of HF, such as HFrEF and HFpEF, which is also a novel aspect.

## Limitations

The major source of limitation is the different follow-up periods of the included studies, ranging from a minimum of 60 days to a maximum of 4.2 years. Furthermore, in some trials, the presence of HF at baseline was assessed by medical history only rather than echocardiographic parameters or biomarkers; hence, there is a chance that some patients with present heart failure were undiagnosed. Majority of the trials examined the most common SGLT2 inhibitors empagliflozin, dapagliflozin, and canagliflozin; for this reason, the cardioprotective results for these were the most prominent compared to those of sotagliflozin and ertugliflozin.

## Conclusion

In conclusion, treatment with SGLT2 inhibitors showed robust results in reducing the incidences of HHF, CV death, and all-cause mortality in patients with underlying HF. Furthermore, the SGLT2 inhibitors appear to show a class effect.

## Data Availability Statement

The original contributions presented in the study are included in the article/Supplementary Material, further inquiries can be directed to the corresponding author/s.

## Author Contributions

GMG and JMSM conceptualized the study and were involved in the acquisition and interpretation of the data. GMG, GG, and JMSM performed the statistical analysis. GMG drafted the manuscript. JMSM is the guarantor of the article. All authors were substantially involved in the critical revision of the manuscript, read, and approved the final version of the manuscript.

## Conflict of Interest

DL has unrestricted research grants from Boehringer Ingelheim, MSD, and Novartis; is on the Speakers' Bureau of Astra Zeneca, Bayer, Boehringer Ingelheim, and NovoNordisk; and is an advisor for Bayer. HS has unrestricted research grants from Boehringer Ingelheim, Eli Lilly, MSD, NovoNordisk, and Sanofi and is on the Speakers' Bureau of AstraZeneca, Amgen, Boehringer Ingelheim, BMS, Eli Lilly, NovoNordisk, Novartis, and Sanofi. JMSM received lecture fees from Bayer, Daiichi, Chiesi and BMS, unrelated to this publication. The remaining authors declare that the research was conducted in the absence of any commercial or financial relationships that could be construed as a potential conflict of interest.

## Abbreviations

SGLT2: sodium-glucose co-transporter 2
T2DM: type 2 diabetes mellitus
HF: heart failure
HHF: hospitalization for heart failure
CV: cardiovascular
RCT: randomized controlled trial
RR: relative risk
RRR: relative risk reduction
ARR: absolute risk reduction
EF: ejection fraction
HFpEF: HF with preserved ejection fraction
HFmrEF: HF with mid-range ejection fraction
HFrEF: HF with reduced ejection fraction
ARNI: angiotensin receptor neprilysin inhibitor
MRA: mineralocorticoid receptor antagonist
NNT: number needed to treat
CI: confidence interval
HR: hazard ratio
eGFR: estimated glomerular filtration rate
BMI: body mass index.
