# Supplementary material for: Cardiovascular Outcome in Patients Treated With SGLT2 Inhibitors for Heart Failure: A Meta-Analysis
Source: Front Cardiovasc Med. 2021 Jul 14;8:691907. doi: 10.3389/fcvm.2021.691907 (PMC8316592; doi:10.3389/fcvm.2021.691907)
Supplement: Supplementary file 1 [file Data_Sheet_1.docx]

** Figure S1.**

**Figure S2.**

**Figure S3.** Subgroup analyses for the composite outcome according to the period of follow-up.
